# Genome, transcriptome, and metabolome analyses provide new insights into the resource development in an edible fungus *Dictyophora indusiata*

**DOI:** 10.3389/fmicb.2023.1137159

**Published:** 2023-02-09

**Authors:** Mingzheng Duan, Shengfeng Long, Xiaojian Wu, Bin Feng, Sunqian Qin, Yijie Li, Xiang Li, Changning Li, Chenggang Zhao, Lingqiang Wang, Yong Yan, Jianming Wu, Falin Zhao, Zhendong Chen, Zeping Wang

**Affiliations:** ^1^Sugarcane Research Institute, Guangxi Academy of Agricultural Sciences/Sugarcane Research Center, Chinese Academy of Agricultural Science/Key Laboratory of Sugarcane Biotechnology and Genetic Improvement (Guangxi), Ministry of Agriculture, Nanning, China; ^2^Key Laboratory for Conservation and Utilization of Subtropical Agro-Bioresources, College of Agriculture, Guangxi University, Nanning, China; ^3^Guangxi Academy of Agricultural Sciences, Nanning, China; ^4^Laibin Branch of Guangxi Academy of Agricultural Sciences, Laihua Center, Laibin, China

**Keywords:** widely-targeted metabolome, food chemistry, edible fungi, tryptophan, UPLC-ESI-MS/MS

## Abstract

*Dictyophora indusiata* (Vent. Ex Pers.) Fisch. (DI) is an edible and medicinal fungus widely used in East Asian countries. However, during DI cultivation, the formation of fruiting bodies cannot be regulated, which leads to yield and quality losses. The present study performed a combined genome, transcriptome, and metabolome analysis of DI. Using Nanopore and Illumina sequencing approaches, we created the DI reference genome, which was 67.32 Mb long with 323 contigs. We identified 19,909 coding genes on this genome, of which 46 gene clusters were related to terpenoid synthesis. Subsequent transcriptome sequencing using five DI tissues (cap, indusia, mycelia, stipe, and volva) showed high expression levels of genes in the cap, indicating the tissue’s importance in regulating the fruiting body formation. Meanwhile, the metabolome analysis identified 728 metabolites from the five tissues. Mycelium was rich in choline, while volva was rich in dendronobilin; stipe had monosaccharides as the primary component, and the cap was the main source of indole acetic acid (IAA) synthesis. We confirmed the importance of tryptophan metabolism for DI fruiting body differentiation based on KEGG pathway analysis. Finally, the combined multiomics analysis identified three new genes related to IAA synthesis of the tryptophan metabolic pathway in the cap, which may regulate DI fruiting body synthesis and improve DI quality. Thus, the study’s findings expand our understanding of resource development and the molecular mechanisms underlying DI development and differentiation. However, the current genome is still a rough draft that needs to be strengthened.

## 1. Introduction

Edible and medicinal fungi are known for their nutritional and medicinal values and are consumed by people worldwide (Buckley, 2008; Lu et al., 2020). The edible and medicinal fungi promote human health and are considered functional foods (Hetland et al., 2008; Li et al., 2019; Krakowska et al., 2020). Among the many edible and medicinal fungi distributed worldwide, *Dictyophora indusiata* (DI, Phallaceae) is widely used in East Asian countries as both medicine and food (Wang et al., 2021). Currently, DI is known for two primary uses; the stipe is used as a delicacy and has various medicinal properties, such as antitumor effects, eye benefits, lipofuscin resistance, cardiovascular protective, antibiosis, mental tranquilization, immunomodulatory, and antioxidant activities (Liao et al., 2015; Liu et al., 2017; Wang Y. et al., 2019; Wang W. et al., 2019). It is also a good source of fungal polysaccharides (Liu et al., 2017; Wang W. et al., 2019) and bioactive compounds (Huang et al., 2011), which enhance human health.

**Figure 1:**
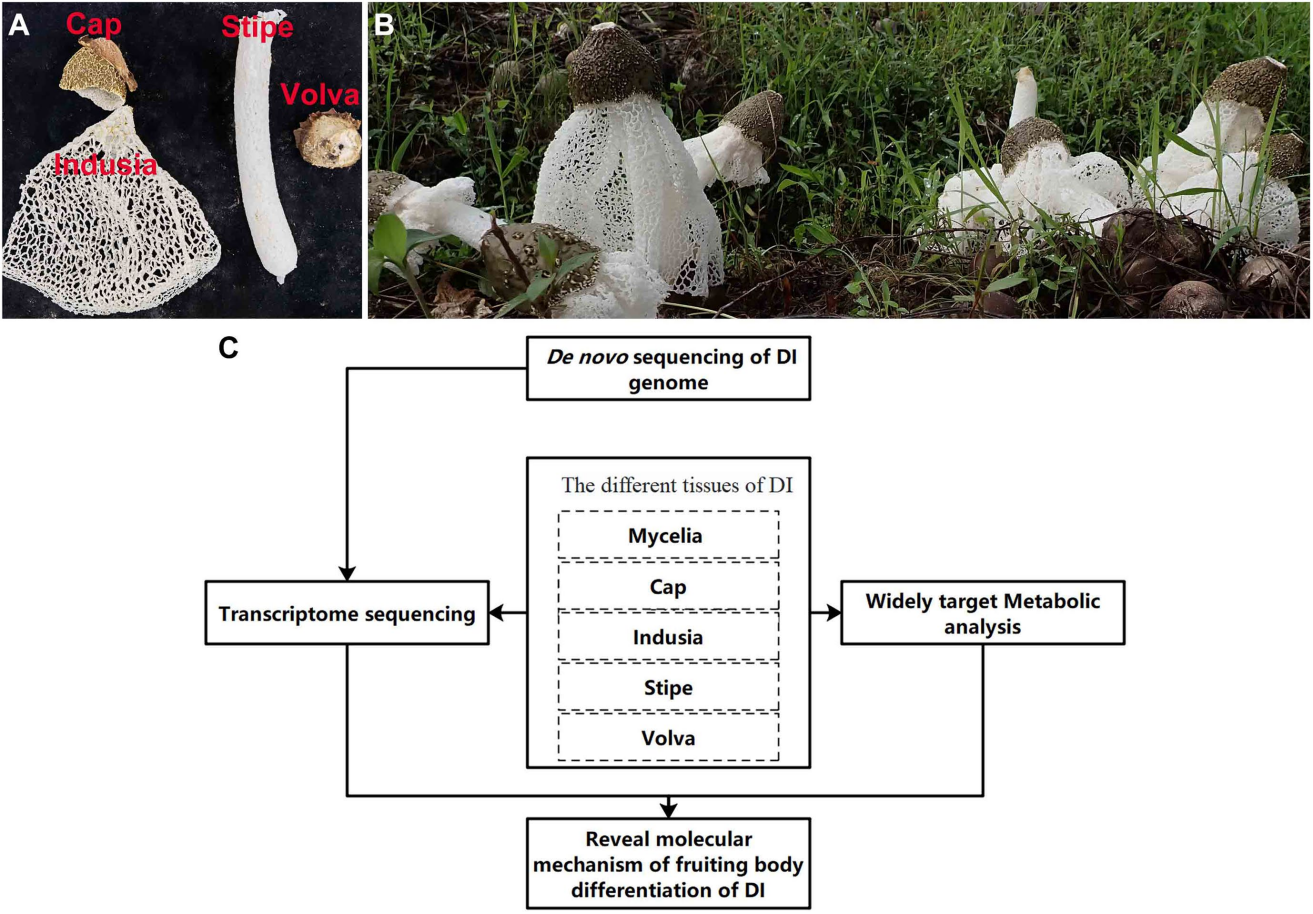
Apparent morphology of DI fruiting bodies and technical route of the study. **(A)** Different kinds of DI tissues used in this study. **(B)** Morphology of DI fruiting bodies at harvest. **(C)** The technical route of this study.

DI has been domesticated and produced artificially in the south of China; however, there are many problems in its cultivation, resource utilization, and development. The short mature stage (few hours) of DI fruiting bodies demands prompt harvesting; delayed harvest causes autolysis of fruiting bodies, leading to wastage. The mature fruiting bodies comprise four tissues (Figure 1A). In south China, usually only the stipe has been consume, while the other three tissues (cap, indusia, and volva) get discarded, which leading to a considerable waste of resources. Wang et al. (2020, 2021), based on the transcriptome, proteome, and metabolome analyses, showed that the cell wall stress-dependent MAPK pathway and a few other unique proteins and metabolites play critical roles in the morphological development of DI. However, the formation of DI fruiting bodies is not entirely explored due to the lack of a reference genome. Meanwhile, the crucial tissue regulating fruiting body formation remains unclear, and the DI mycelium remains less exploited for medicinal use. Moreover, DI has various bioactive compounds, and the genome harbors numerous functional genes related to metabolites with medicinal properties; however, research in this field is less. Therefore, addressing these issues and improving our understanding will promote the economic value of DI.

In recent years, advanced omics technologies have been applied to study fungi. A combination of second-generation and third-generation sequencing technologies has been widely used to analyze the genome of edible and medicinal fungi, such as *Russula griseocarnosa*, *Agrocybe cylindracea*, *Hericium erinaceus*, *Auricularia heimuer*, and *Gloeostereum incarnatum* (Wang X. et al., 2019; Yuan et al., 2019; Gong et al., 2020; Liang et al., 2020; Yu et al., 2020). Transcriptomic studies revealed the fruiting body formation of edible and medicinal fungi, such as *Leucocalocybe mongolica*, *Lentinula edodes*, and the peach-shaped and mature fruiting bodies of DI (Yoo et al., 2019; Wang et al., 2020; Duan et al., 2021). Meanwhile, metabolomic studies characterized the edible and medicinal fungi and their diverse medicinal components, such as bioactive metabolites, antibiotics, and agrochemicals (Alberti et al., 2020). These earlier studies indicated that combining various “omics” technologies would help elucidate DI fruiting body differentiation and explore resource value.

Therefore, the present study investigated the molecular mechanism underlying DI fruiting body differentiation and the resource value of medicinal compounds and gene resources based on sequencing and UHPLC-ESI-MS/MS. We present a reference genome of DI based on third-generation sequencing. Then, transcriptome sequencing was performed using five DI tissues (cap, indusia, mycelia, stipe, and volva) to identify the tissues with the most significant influence on fruiting body differentiation. Further, a widely-targeted metabolome analysis was performed to explore the medicinal value of these five DI tissues and the metabolic processes affecting the DI fruiting body differentiation. Finally, the functional gene resources associated with the high value metabolites in the DI genome were mined based on multi-omics association analysis. The findings of the study will lay a foundation for improving the quality and resource value of DI.

## 2. Results

### 2.1. *De novo* genome sequencing

#### 2.1.1. Reference genome of *Dictyophora* genus

We constructed the reference genome of DI to advance our understanding of the molecular mechanisms underlying DI fruiting body development and differentiation. As shown in Table 1, third-generation nanopore sequencing combined with NGS sequencing generated a reference genome of 67.32 Mb (Strain ID: ZS). The genome had 216 contigs, a GC content of 44.05%, a contig N50 of 0.79 Mb, L50 of 20, and 19,909 genes. BUSCO analysis recovered 87.6% (254/290) of the core genes (Supplementary Table S1), indicating high integrity of the assembled genome.

**Table 1:** Statistics of assembly of the DI genome.

Genome ID	ZS
Genome assembly size (Mb)	67.32
GC content (%)	44.05
Contigs	216
N50 (Mb)	0.79
L50	20
Largest contig (Mb)	3.39
Gene number	19,909
Nanopore sequencing coverage	181×
NGS sequencing coverage	176×

#### 2.1.2. Genome annotation based on public databases

**Figure 2:**
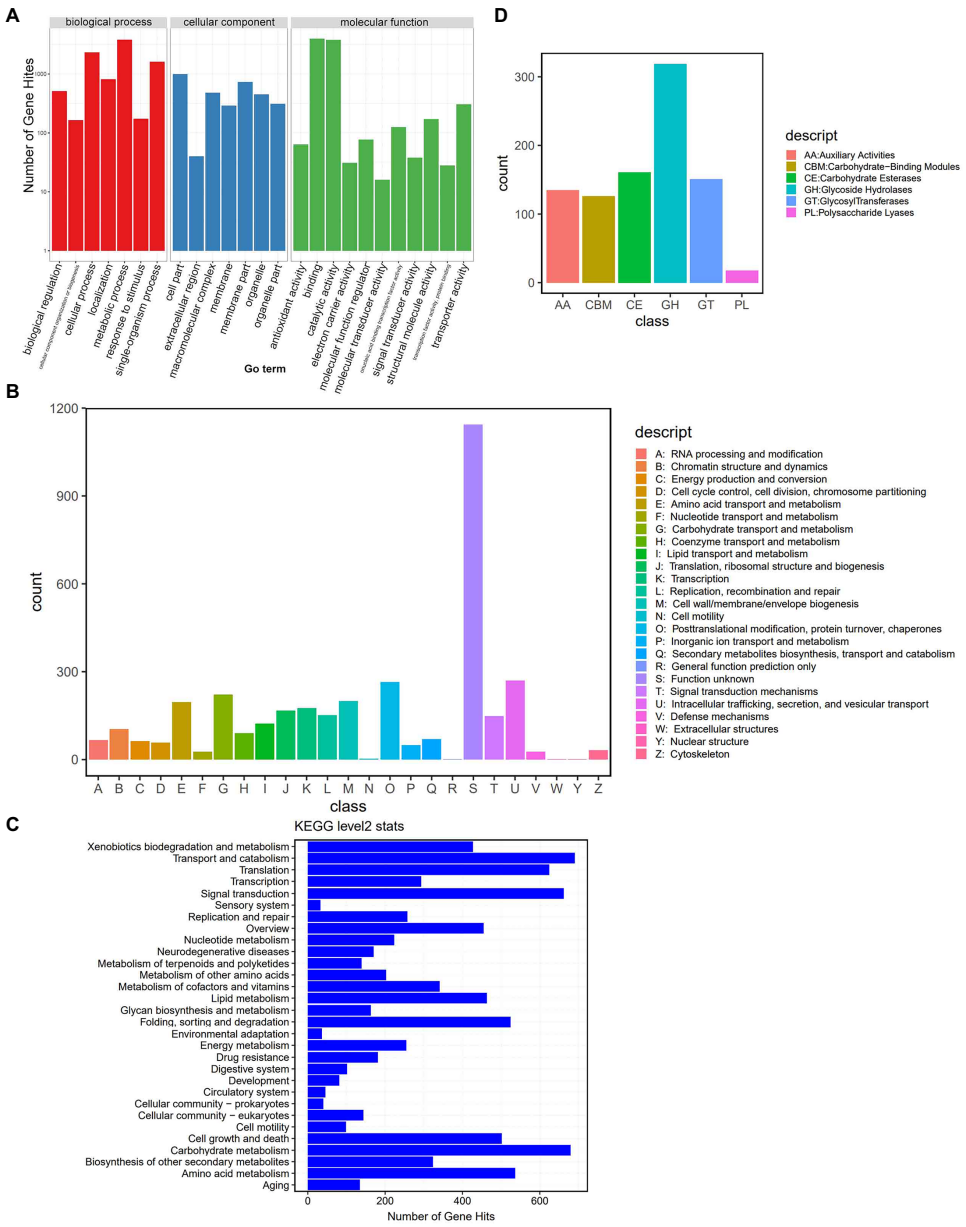
Genome annotation based on public databases. **(A)** GO annotation. The upper horizontal axis shows the three GO categories (Biological Process – BP, Cellular Component – CC, and Molecular Function – MF), the lower horizontal axis shows the subclassification of these three categories, and the vertical axis represents the number of gene hits. **(B)** KOG annotation. The horizontal axis shows the KOG classes, and the vertical axis represents the number of gene hits. **(C)** KEGG (Level 2) annotation. The horizontal axis represents the number of gene hits, and the vertical axis shows the classes. **(D)** CAZyme annotation. The horizontal axis shows the classes, and the vertical axis represents the number of gene hits.

We further annotated the DI genes using public databases. The predicted 19,909 genes of DI (Strain ZS; a total length of 31.88 Mb, 47.35% of the genomic size) showed an average gene length of 1,601 bp and an average GC content of 47.25%. We annotated these genes using the four public databases, GO (Gene Ontology), KOG (Eukaryotic Orthologous Groups), KEGG (Kyoto Encyclopedia of Genes and Genomes), and CAZyme (Carbohydrate-Active enzymes). The preliminary comparison showed the annotation of 39.63% (7,891), 16.98% (3,381), 51.22% (10199), and 4.14% (826) of genes in the GO, KOG, KEGG, and CAZyme databases, respectively. In the GO categories (Figure 2A), 3,815 genes enriched “metabolic process,” the predominant term in the biological process category; 999 genes enriched “cell part,” the predominant term in the cellular component category; 3,975 genes enriched “binding,” the predominant term in the molecular function category. KOG annotation (Figure 2B) assigned 1,143 genes to the code class “S: Function unknown,” which was the predominant class. The second most enriched code class was “U: Intracellular trafficking, secretion, and vesicular transport,” with 270 genes. Meanwhile, KEGG annotation (Level 2) predominantly enriched “Transport and catabolism” with 690 genes (Figure 2C). The annotation based on CAZyme divided the genes into 910 gene families (Figure 2D), of which 135 belonged to AA (Auxiliary Activities), 126 to CBM (Carbohydrate-Binding Modules), 161 to CE (Carbohydrate Esterases), 319 to GH (Glycoside Hydrolases), 151 to GT (Glycosyl Transferases), and 18 to PL (Polysaccharide Lyases).

#### 2.1.3. Secondary metabolism-associated genes of DI

Further annotations based on FCPD (Fungal cytochrome P450 database) and antiSMASH revealed genes associated with the secondary metabolites of DI. The FCPD pipeline identified 369 DI genes related to 41 of P450 gene families (Supplementary Table S2). Meanwhile, antiSMASH showed that 64 gene clusters of DI were associated with secondary metabolism synthesis (Supplementary Table S3). Among them, 64 gene clusters were related to the terpene class, and the remaining were associated with the synthesis of indole, T1PKS (Type I PKS: Polyketide synthase), NRPS (Non-ribosomal peptide synthetase cluster), siderophore, and NRPS-like (NRPS-like fragment) metabolites.

### 2.2. Transcriptome sequencing of five DI tissues

#### 2.2.1. Sequencing analysis and differentially expressed genes (DEGs)

Further, to reveal the differences in gene expression patterns between different tissue types of DI, transcriptome sequencing was performed using different fruiting body tissues (cap, indusium, mycelium, stipe, and volva) and the dikaryotic mycelium as control. As shown in Table 2, a total of 109.15 GB of NGS data were generated, with an average of 6 GB per sample; the lowest Q30 value was 91.87% (Stipe3), and the total mapped rate of all samples was at least 95.8% (Volva1), indicating the high quality of the transcriptome data.

**Table 2:** Statistics of the transcriptome sequences from five DI tissues.

ID	Clean Data (GB)	Q30 (%)	Total Mapped (%)
Cap1	6.64	92.85%	96.36%
Cap2	6.41	92.61%	96.18%
Cap3	6.52	92.60%	96.42%
Indusia1	7.76	92.24%	95.93%
Indusia2	7.51	92.55%	96.46%
Indusia3	7.38	93.02%	96.39%
Mycelia1	6.97	92.44%	96.89%
Mycelia2	7.78	93.19%	97.06%
Mycelia3	6.74	92.62%	96.95%
Stipe1	6.93	93.05%	96.69%
Stipe2	7.75	92.89%	96.46%
Stipe3	7.63	91.87%	96.20%
Volva1	7.55	92.54%	95.80%
Volva2	7.51	92.64%	96.29%
Volva3	7.99	92.38%	96.08%

**Figure 3:**
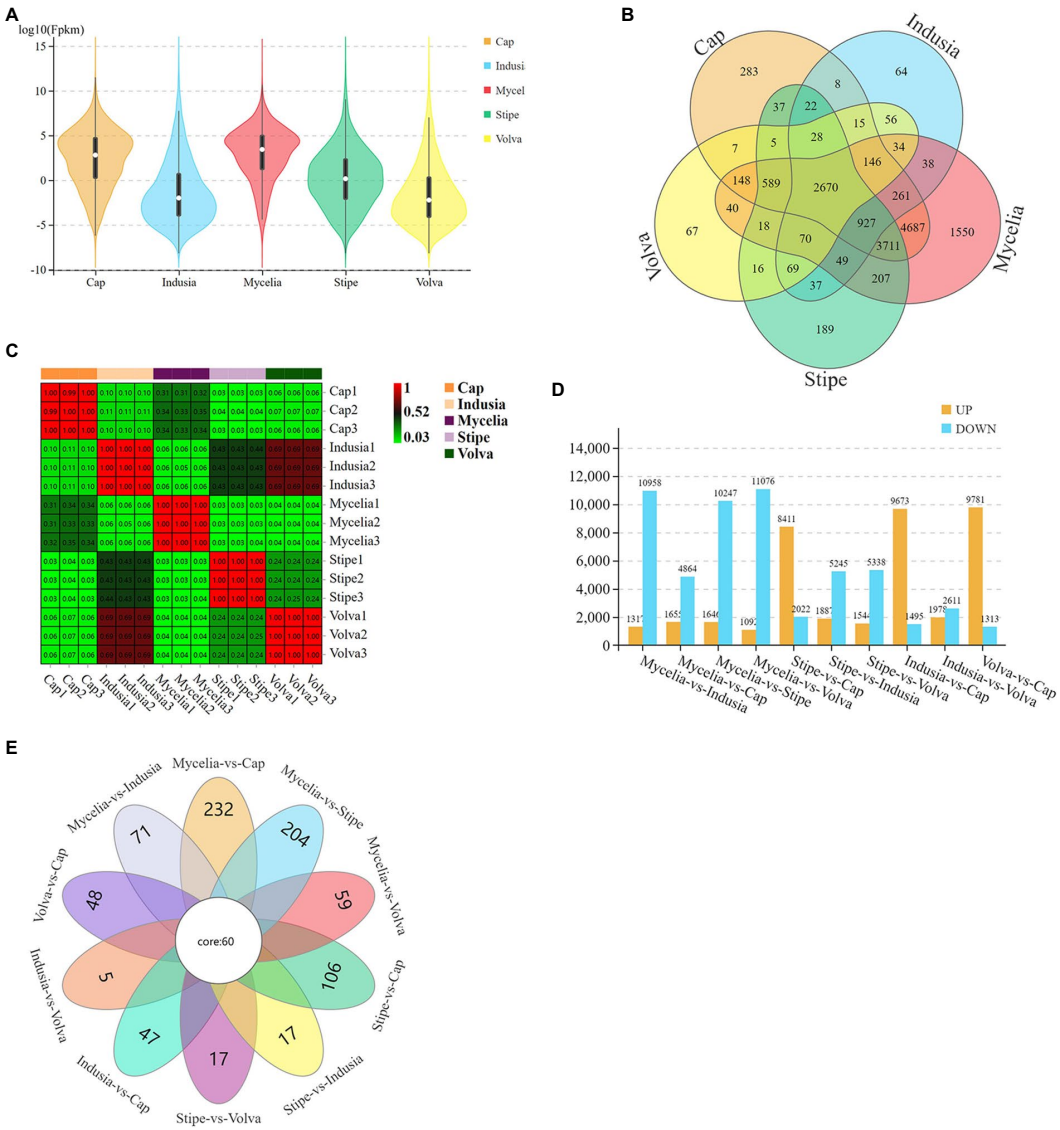
Distribution of DEGs across different DI tissues. **(A)** The violin plots display the gene expression intensity in five tissue types of DI. The curves represent the probability curve of the data distribution. The number of data points is positively correlated with the width of the probability curve. The upper and lower ends indicate the maximum and minimum values of nonoutliers, respectively. The upper and lower edges of the vertical line indicate the 75^th^ and 25^th^ percentiles of the data, respectively; the central dot indicates the median. The horizontal axis represents different tissues, and the vertical axis represents the log_10_FPKM. **(B)** Venn diagram of DEGs in five tissues. Different colors represent different tissues. **(C)** Correlation heat map based on the expression of DEGs in five tissues. The abscissa and ordinate indicate the samples. The numbers in the grid are the Pearson correlation coefficient values. The different colors of the grid indicate different correlations. Red means a positive correlation; the darker the color, the stronger the correlation. **(D)** DEGs of each comparison group. The abscissa represents different comparison groups, and the ordinate represents the number of DEGs. **(E)** Venn diagram of DEGs of different comparison groups.

We then identified the DEGs (Figure 3). The violin plots showed higher expression levels of genes in cap and mycelia but lower in indusia and volva (Figure 3A); the expression of genes was moderate in the stipe. Venn diagram showed that 2,670 DEGs were shared between all tissues, while 4,687 were common between mycelia and cap; 64, 67, 189, 283, and 1,550 DEGs were unique to the indusia, volva, stipe, cap, and mycelia (Figure 3B). The correlation heat map showed high association in the expression pattern of DEGs between indusia and volva (*R* = 0.69; Figure 3C). The subsequent pairwise comparison of tissues revealed two expression patterns of DEGs (Figure 3D); one group showed significantly downregulated expression in all comparisons with mycelia (mycelia vs. indusia, cap, stipe, and volva; e.g., mycelia vs. indusia with 10,958 and 1,317 of downregulated and upregulated DEGs); another group had DEGs upregulated in cap compared with other tissues, except mycelia (stipe, indusia, and volva vs. cap). Another Venn diagram based on DEGs of different comparison groups showed that unique DEGs related to mycelia and cap were significantly higher than those between other tissues; mycelia vs. cap had 232 unique DEGs, but indusia vs. volva had only 5 unique DEGs (Figure 3E). Thus, the observations indicated that among all the five DI tissues, mycelium and cap were the most significantly active tissues with numerous DEGs, predominantly upregulated genes, suggesting their leading role in the differentiation of DI fruiting bodies; this confirmed mycelium and cap as critical tissues for the differentiation of DI fruiting bodies.

#### 2.2.2. Go and KEGG differential genes enrichment analysis

**Figure 4:**
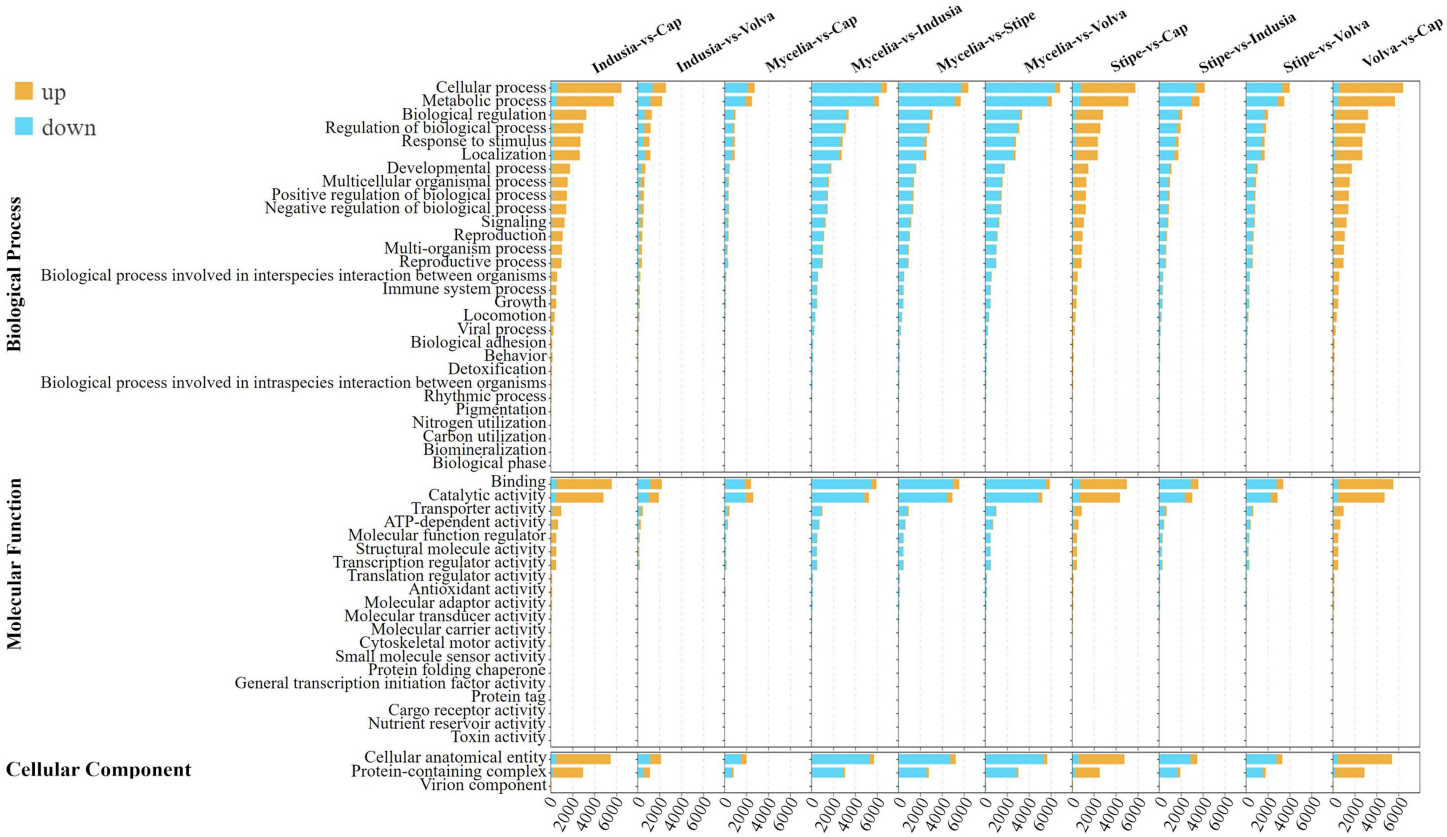
GO enrichment of the differentially expressed genes of different comparison groups. Different colors indicate the number of upregulated and downregulated genes in multiple comparison groups. In the upper left corner is the figure legend.

To further determine the functions of these DEGs, we performed enrichment analysis based on GO and KEGG databases (Figure 4; Supplementary Figure S1). GO annotation showed that “cellular process (GO:0009987),” “metabolic process (GO:0008152),” and “biological regulation (GO:0065007)” were the top three enriched terms in the biological process category; “binding (GO:0005488)” and “catalytic activity (GO:0003824)” were the two significantly enriched terms in molecular functions; “cellular anatomical entity (GO:0110165)” and “protein-containing complex (GO:0032991)” were the two significantly enriched terms in cellular component (Figure 4). Meanwhile, KEGG enrichment analysis showed global and overview map (ko01100) as the most significantly enriched pathway in all comparison groups and level 1 class of metabolism (Supplementary Figure S1). In the indusia vs. cap comparison, the Metabolic pathway (ko01100) had 1,538 DEGs, while the translation (ko00970) pathway was enriched with 462 DEGs. Meanwhile, the translation (ko00970), transport and catabolism (ko04138), signal transduction (ko04011), and aging (ko04213) pathways were the most enriched in the level 1 class of Genetic In-formation Processing, Cellular Processes, Environmental Information Processing, and Organismal Systems. We found that the DEGs expression trend in the above two databases was consistent with distribution of DEGs across different DI tissues (Figure 3), indicating the reliability of the annotation.

### 2.3. Widely-targeted metabolome analysis of five DI tissue

#### 2.3.1. Metabolite composition

**Figure 5:**
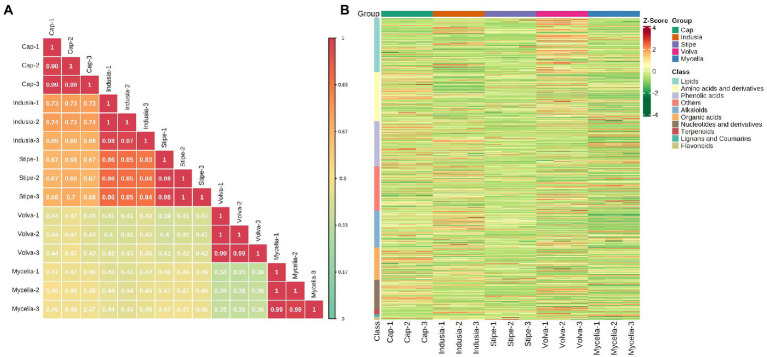
Metabolite composition of five DI tissues. **(A)** Pearson’s correlation heat map. The longitudinal and diagonal lines show the sample names, and different colors represent the different Pearson correlation coefficients; the key is shown on the right side. **(B)** Clustering heat map of all metabolites. Each column represents a sample, and each row represents a metabolite class. A color bar represents the metabolite abundance. Different shades of red and green represent the upregulated and downregulated metabolites, respectively.

We performed a widely-targeted metabolome analysis based on the UPLC-ESI-MS/MS approach to determine the metabolic composition of different DI tissues. We determined the Z-scores to evaluate the categories and abundance of metabolites (Figure 5) and then analyzed the sample-sample correlation of metabolite distribution in the five tissues. As shown in Figure 5A, the cap, indusia, and stipe metabolites were highly correlated (R > 0.67). Further, based on public and commercial databases, 728 metabolites (grouped into ten classes), including 89 of alkaloids, 119 of amino acids and derivatives, 6 of flavonoids, 7 of lignans and coumarins, 130 of lipids, 71 of nucleotides and derivatives, 78 of organic acids, 109 of phenolic acids, 12 of terpenoids, and 107 of metabolites belong to others, were detected in the five DI tissues (Figure 5B). Detailed information on the metabolites is presented in Supplementary Table S4.

#### 2.3.2. Differential metabolites

**Figure 6:**
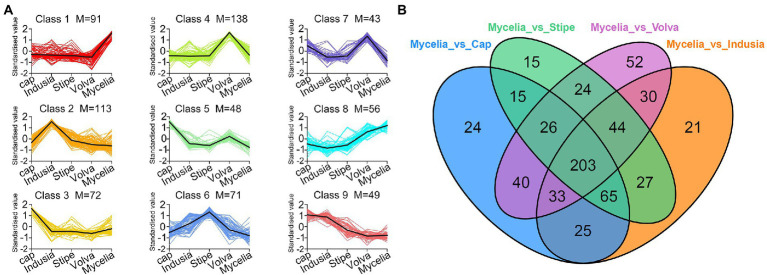
Analysis of the distribution characteristics of metabolites in different DI tissue types. **(A)** K-means clustering of metabolites of five DI tissue types. The abscissa represents the sample group, the ordinate represents standardized relative metabolite content, and the class represents the metabolite category number with the same trend in five DI tissue types. M represents the number of metabolites in this class. **(B)** Venn diagram shows differential metabolites of different comparison groups.

Furthermore, we compared the metabolite abundance among the five tissues and analyzed their distribution characteristics. K-means clustering was performed to group the metabolites based on the abundance in the different tissues (Figure 6A; Supplementary Table S5). A total of nine groups of metabolites were identified with specific expression trends. We identified 91 metabolites in mycelia (class 1), 138 in volva (class 4), 113 in indusia (class 2), 72 in cap (class 3), and 71 in stipe (class 6) with representative of the tissue types. Further, we analyzed the most abundant tissue-specific metabolites (Table 3). Choline, dibutyl phthalate*, and diisobutyl phthalate* were the top three tissue-specific metabolites in mycelia, 13(S)-HODE;13(S)-hydroxyoctadeca-9Z,11E-dienoic acid, 9S-Hydroxy-10E,12Z-octadecadienoic acid, and punicic acid (9Z,11E,13Z-octadecatrienoic acid) in volva, linoleic acid, D-Pantothenic acid, and naphthisoxazol A in indusial, succinyladenosine, isocitric acid, and quinic acid in the cap, and D-mannose*, inositol*, and D-glucose* in stipe. Furthermore, we analyzed the relationship between the differential metabolites in each tissue compared with mycelia to screen metabolites related to DI fruiting body differentiation. As shown in Figure 6B, mycelia vs. volva (52) had the maximum unique, differential metabolites compared with mycelia vs. cap (24), mycelia vs. stipe (15), and mycelia vs. indusia (21).

**Table 3:** Top three metabolites in the DI tissues.

Tissue	Class	Formula	Compounds	Class type
Mycelia	1	C_5_H_14_NO^+^	Choline	Alkaloids
	1	C_16_H_22_O_4_	Dibutyl phthalate*	Phenolic acids
	1	C_16_H_22_O_4_	Diisobutyl phthalate*	Phenolic acids
Volva	4	C_18_H_32_O_3_	13(S)-HODE;13(S)-Hydroxyoctadeca-9Z,11E-dienoic acid	Lipids
	4	C_18_H_32_O_3_	9S-Hydroxy-10E,12Z-octadecadienoic acid	Lipids
	4	C_18_H_30_O_2_	Punicic acid (9Z,11E,13Z-octadecatrienoic acid)	Lipids
Indusia	2	C_18_H_32_O_2_	Linoleic acid	Lipids
	2	C_9_H_17_NO_5_	D-Pantothenic Acid	Others
	2	C_11_H_9_NO_2_	Naphthisoxazol A	Alkaloids
Cap	3	C_14_H_17_N_5_O_8_	Succinyladenosine	Nucleotides and derivatives
	3	C_6_H_8_O_7_	Isocitric Acid	Organic acids
	3	C_7_H_12_O_6_	Quinic Acid	Organic acids
Stipe	6	C_6_H_12_O_6_	D-Mannose*	Others
	6	C_6_H_12_O_6_	Inositol*	Others
	6	C_6_H_12_O_6_	D-Glucose*	Others

*Indicates the metabolite presence of isomer.

**Figure 7:**
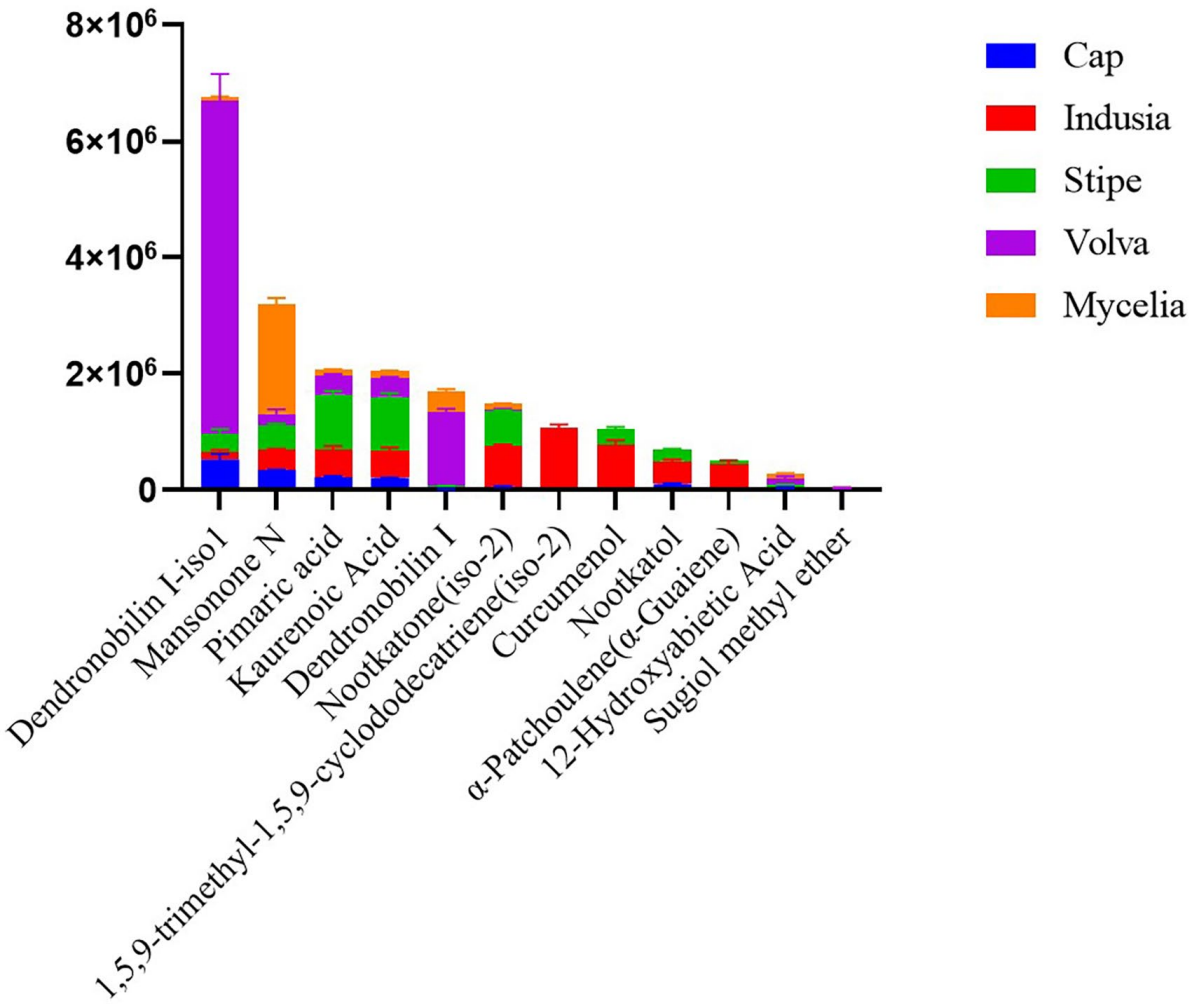
Distribution of terpenoids in different DI tissues. The abscissa represents the metabolite name, and the ordinate represents the peak area unit.

We then analyzed the distribution of terpenoid metabolites in DI in detail. As shown in Figure 7, 12 terpenoids were detected from the five DI tissue types. Among them, Dendronobilin I-iso1 and Mansonone N were the top two terpenoids in terms of intensity, with Dendronobilin I-iso1 mainly synthesized in volva and Mansonone N in mycelia.

#### 2.3.3. Tryptophan metabolism of DI

**Figure 8:**
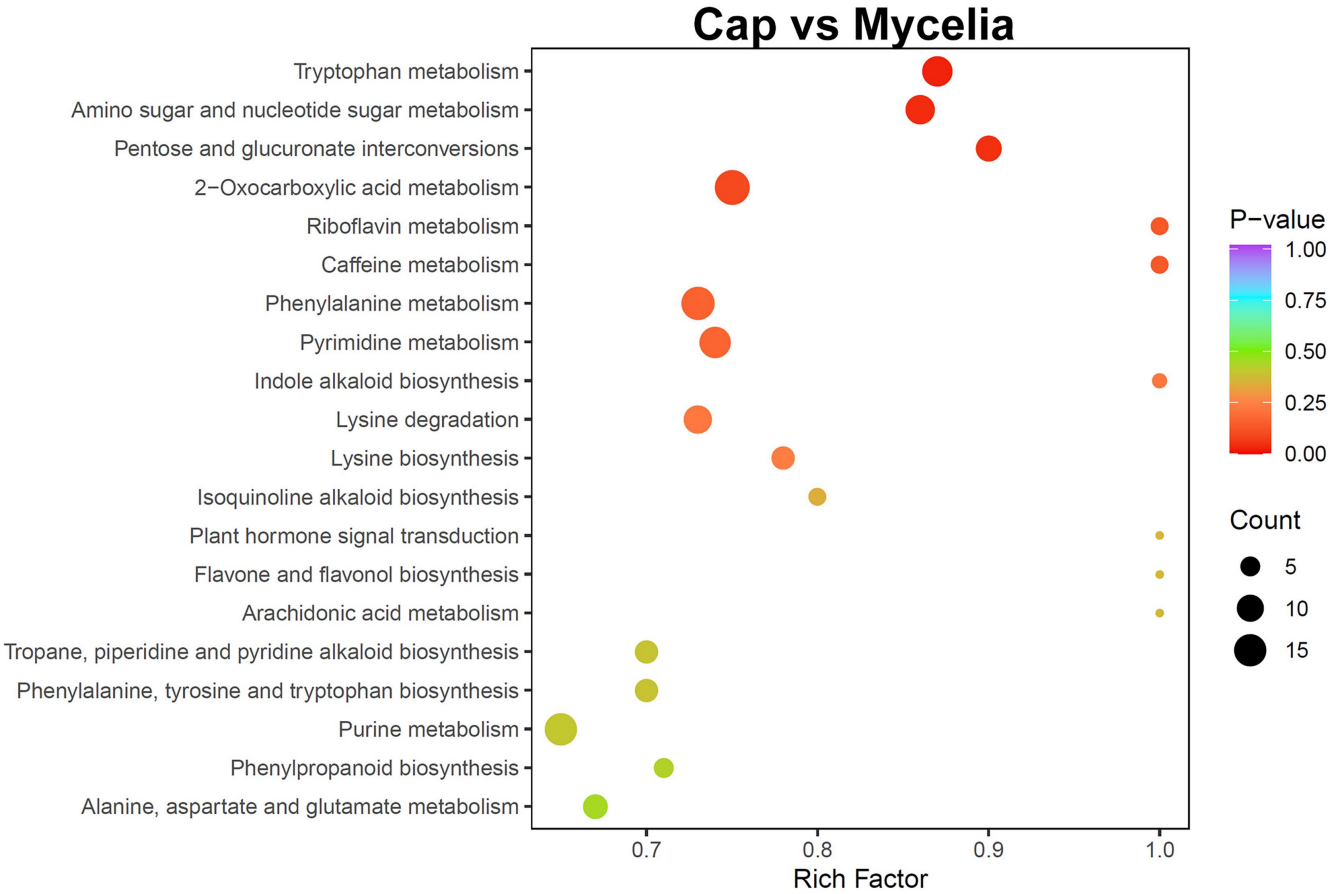
KEGG pathway enrichment analysis of the metabolome between cap and mycelia. The abscissa represents the rich factor of each pathway, and the ordinate represents the pathway name (sorted by value of *p*). The color of the dots reflects the value of *p*; the redder the dots, the more significant the enrichment. The dot size represents the number of differential metabolites enriched in the pathway.

**Figure 9:**
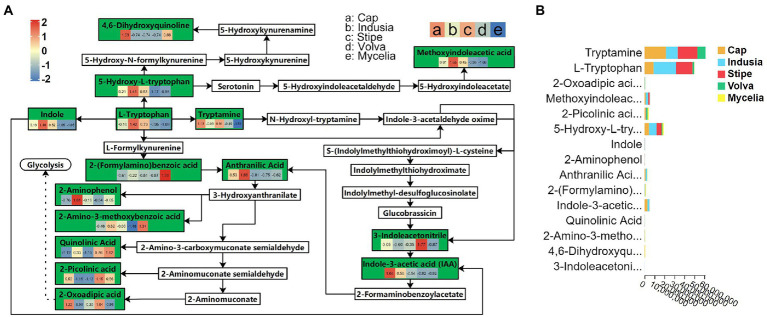
Tryptophan metabolism pathway (ko00380) in five DI tissues. **(A)** The boxes represent metabolites involved in the pathway. Green boxes represent the detected metabolites, and white boxes represent the undetected metabolites. The color (red to blue) key in the green boxes represents the relative abundance of the metabolite in different tissues (Evaluated by *z*-value, the key is shown in the upper left corner). The alphabets used for the tissues are shown in the upper right corner. The solid arrows represent the metabolic process, and the dashed arrows leading to glycolysis represent the processes in which some metabolites are ignored; metabolites eventually end up in glycolysis. **(B)** Relative abundance of metabolites of the ko00380 pathway in different DI tissues. The abscissa represents the peak area unit of the metabolite, and the ordinate represents different metabolites. Different colors in the stack diagram indicate different tissues shown in the legend in the upper right corner.

Transcriptome analysis detected the highest abundance of DEGs in the cap and mycelia, suggesting that these two tissues were critical for DI fruiting body differentiation. Therefore, we conducted metabolic pathway enrichment analysis for the cap and mycelia based on the KEGG database. As shown in Figure 8, three pathways, including Tryptophan metabolism (ko00380), Amino sugar and nucleotide sugar metabolism (ko00520), and Pentose and glucuronate interconversions (ko00040), were significantly (*p* < 0.05) enriched in the cap vs. mycelia comparison group. We detected the highest enrichment intensity for Tryptophan metabolism, with the highest participation of metabolites. As shown in Figure 9, a large amount of L-Tryptophan was first synthesized and enriched in the cap, indusia, and stipe during mycelium differentiation into fruiting bodies, and most of the L-Tryptophan metabolized to tryptamine (Figures 9A,B). As Figure 9A, through a series of metabolic processes, tryptamine got metabolized to 3-indoleacetonitrile and then indole-3-acetic acid (IAA). Next, IAA got metabolized into a node metabolite anthranilic acid, which further converted into 2-aminophenol, 2-amino-3-methoxybenzoic acid, quinolinic acid, 2-picolinic acid, and 2-oxoadipic acid. Finally, 2-oxoadipic acid got metabolized *via* the next metabolic pathway of glycolysis (ko00010). Thus, these observations indicated that during the formation of DI fruiting bodies from mycelium, the metabolite L-tryptophan and the Tryptophan metabolism pathway play significant roles.

### 2.4. Combined multi-omics analysis

#### 2.4.1. Transcriptome and metabolome correlation analysis

**Figure 10:**
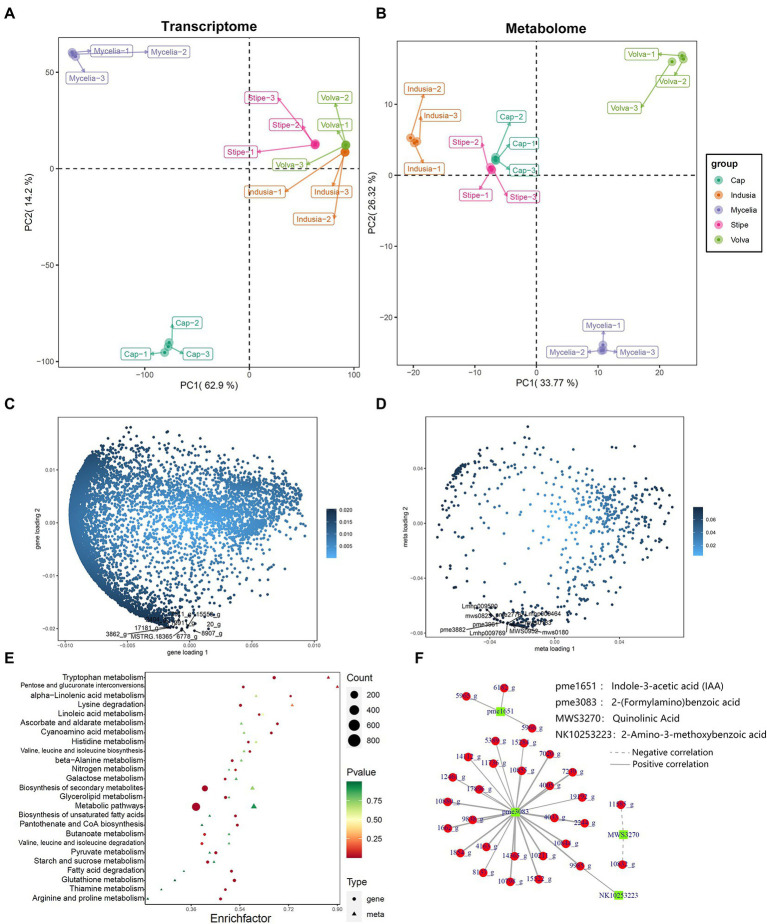
Correlation between metabolome and transcriptome of DI. **(A,B)** Metabolome and transcriptome PCA analysis. The abscissa represents PC1, and the ordinate represents PC2. **(C,D)** Metabolome and transcriptome O2PLS analysis. The abscissa represents the one-dimensional gene **(C)**/metabolite **(D)** loading value, and the ordinate represents the two-dimensional gene **(C)**/metabolite **(D)** loading value. **(E)** KEGG pathway enrichment analysis of DEGs and differential metabolites between cap and mycelia. The abscissa represents the corresponding enrichment factor of each pathway, and the ordinate represents the pathway name (sorted by value of *p*). The color of the dots and the triangles reflects the value of *p* of the genes and corresponding metabolites; the redder the dots/triangle, the more significant the enrichment. The size of the dot/triangle represents the number of differential genes and metabolites. Figure legend is shown on the right side. **(F)** Pearson correlation network between DEGs and metabolites. Red represents DEGs, and the green represents metabolites.

Furthermore, to understand the key genes and metabolites affecting DI fruiting body differentiation, we performed a correlation analysis based on the metabolome and transcriptome data and assessed the metabolite and gene expression similarity among the five tissue types. PCA based on mycelia’s metabolome and transcriptome data showed significant differences; the mycelia appeared separated from the other four tissue types. The transcriptome PCA showed clustering of the stipe, volva, and indusia (Figure 10A); however, the cap appeared separated from these three tissues. The metabolome PCA showed that the volva was separated, while the indusia, cap, and stipe were clustered together (Figure 10B). These results indicated that the metabolite accumulation and the transcriptome expression were not wholly consistent among the different DI tissue types.

We further identified the transcriptome and metabolome elements associated with fruiting body differentiation based on O2PLS analysis. We selected the top 10 genes and metabolites based on the loading value of expression of the DEGs and the differential metabolites (Figures 10C,D). Further annotation and abundance analysis of these genes and metabolites showed that most of these DEGs were homologous to the hypothetical protein from *Clathrus columnatus* and *Sphaerobolus stellatus*, except for MSTRG.18365, which may be involved in the synthesis of Proteophosphoglycan ppg4 (Supplementary Table S6). Meanwhile, the metabolites included four lipids (lysopc 17:1, lysope 17:1, lysope 17:1(2n isomer), and dihydrosphingosine-1-phosphate), three nucleotides and derivatives (2′-deoxyadenosine, 2′-deoxyuridine, and 2′-deoxyinosine), two phenolic acids (3,4-dihydroxybenzoic acid, protocatechuic acid* and 2,5-dihydroxybenzoic acid, gentisic acid*), and one organic acid (3-methyl-2-oxobutanoic acid, Supplementary Table S7).

#### 2.4.2. Identification of tryptophan metabolism-associated genes in DI

We also performed a KEGG association analysis of transcriptome and metabolome data associated with the tryptophan metabolism pathway (ko00380) to identify the DEGs associated with tryptophan metabolism. KEGG enrichment analysis of the genes related to tryptophan metabolism in the cap vs. mycelia group showed that 50 were associated with the synthesis of 13 metabolites (Figure 10E, *p* = 0.02). Further, we classified the association between these 50 DEGs and 13 metabolites based on Pearson’s correlation analysis. as shown in Figure 10F; Supplementary Table S8; 30 DEGs were significantly associated with four metabolites, including 2-(formylamino)benzoic, indole-3-acetic acid (IAA), uinolinic acid, and 2-amino-3-methoxybenzoic acid, of the tryptophan metabolism pathway. NR database-based annotation showed that most DEGs were homologous to the hypothesized proteins of *Clathrus columnatus*, indicating that their gene functions have not been verified. In addition, among DEGs annotated as non-hypothetical proteins, we found that 11736_g (amidase signature do-main-containing protein), 14112_g (aldehyde dehydrogenase domain-containing protein), 14365_g (pyridoxal phosphate-dependent transferase), 17896_g (amidase signature domain-containing protein), 1854_g (pyridoxal phosphate-dependent transferase), 5389_g (FAD-binding domain-containing protein), and 7020_g (L-tyrosine:2-oxoglutarate aminotransferase) may be related to the synthesis of 2-(Formylamino)benzoic acid, and 5965_g (aldehyde dehydrogenase) may be associated with the synthesis of IAA.

#### 2.4.3. Quantitative polymerase chain reaction validation

**Figure 11:**
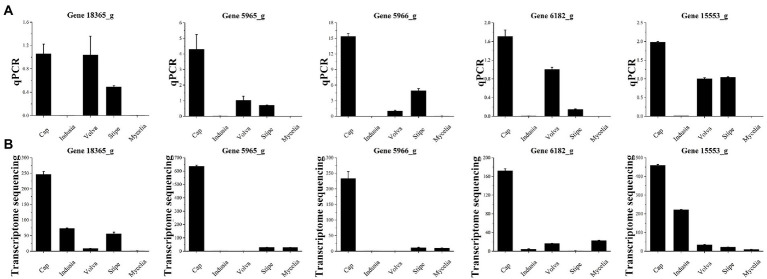
qPCR validation of five genes of five DI tissues identified from transcriptome sequencing. **(A)** Transcriptome sequencing results of the five genes in five DI tissues. The abscissa represents the tissues, and the ordinate represents FPKM expression levels. **(B)** qPCR analysis of the five genes in five DI tissues. The abscissa represents the tissues, and the ordinate represents the 2^-∆∆Ct^ values.

Finally, quantitative polymerase chain reaction (qPCR) (Figure 11) was performed for the five selected genes to verify the accuracy of the results of the combined multi-omics analysis presented in Figure 10. Among the five genes, three (5965_g, 5966_g, and 6182_g) were related to IAA synthesis (Figure 10F; Supplementary Table S8) and two (MSTRG.18365 and 15553_g) were identified based on O2PLS analysis (Figure 10C). We found that four out of the five genes (except for MSTRG.18365) showed an expression trend in qPCR consistent with the transcriptome data, with higher expression in the cap than the other four tissues. These observations indicate that our transcriptome data are reliable.

## 3. Discussion

DI is a delicious edible fungus widely cultivated artificially in east Asia. However, due to the lack of understanding of the molecular mechanisms underlying fruiting body formation, its cultivation and breeding are limited. Moreover, in many regions, such as south China, all DI fruiting bodies are discarded during harvest except the stipe, leading to largescale waste of resources. Therefore, the present study investigated the molecular background and metabolic process of DI fruiting body differentiation.

### 3.1. Genome of DI

Initially, we combined third-generation sequencing with high-throughput sequencing and assembled the DI reference genome, which could be used to understand the molecular details of DI. However, due to dualnucleated strain sequencing, the assembly was not ideal (N50 < 1 Mb). In contrast, recent studies that used similar sequencing strategies for mononuclear edible strains, such as *Leucocalocybe mongolica* (Duan et al., 2021), *Hericium erinaceus* (Gong et al., 2020), *Auricularia heimuer* (Yuan et al., 2019), and *Cordyceps guangdongensis* (Zhang et al., 2018), published genomes with N50 values above 1 Mb. Meanwhile, our assembly results are similar to the genome generated for *Russula griseocarnosa* (Yu et al., 2020) and *Agrocybe cylindracea* (Liang et al., 2020) based on third-generation sequencing and from dikaryotic mycelium or fruiting body (N50 < 1 Mb). Therefore, the present study still provides a reference genome of DI to assemble high quality genomes and data for understanding the developmental details. These observations suggest mononuclear diploid sequencing to obtain a more accurate reference genome. Taken together, the current genome is still a rough draft that needs to be strengthened. Therefore, we plan to further improve the assembly quality and gene annotation level of DI genomes by mononuclear sequencing in the future.

We further predicted 19,909 genes from the DI genome. Due to the largescale diversity in fungal species, our results of predicted genes did not match well with the public databases. Only a small fraction of predicted genes (39.63%) was annotated based on the GO database, consistent with the low annotation rate reported for other edible fungi, such as *Leucocalocybe mongolica* (65.98%) (Duan et al., 2021) and *Russula griseocarnosa* (22.63%) (Yu et al., 2020). These observations indicate space for future research on edible fungi, including DI. Further detailed analysis based on FCPD and antiSMASH annotations showed that 369 genes belonged to the P450 family and 64 belonged to the secondary metabolism gene cluster related to synthesizing secondary metabolites. These secondary metabolites, including the terpenes, have high medicinal value (Muszyńska et al., 2018), indicating significant potential for their application. Moreover, the genes associated with secondary metabolites have been annotated in the genomes of other edible fungi, including *Russula griseocarnosa* (Yu et al., 2020) and *Auricularia heimuer* (Yuan et al., 2019). Therefore, our study provides insights into the potential ability of DI to synthesize terpenes and a genetic basis for the biosynthesis of medicinal compounds.

### 3.2. Transcriptome sequencing reveals cap as critical tissues for DI fruiting body differentiation

In DI transcriptome studies, selecting an appropriate tissue representing the fruiting body is often challenging as the structure of the DI fruiting body is complex. Therefore, in this study, we divided DI into four tissue types. Moreover, our transcriptome study (section 3.2) proved that DEGs varied greatly among the different tissue types and identified a representative tissue, Cap, for DI fruiting body studies.

Our study found that the expression intensity of the DEGs in the comparison groups associated with cap or mycelia were significantly higher than the other tissues (Figure 3A), and these groups had significantly more common DEGs than the other groups (Figure 3B); the comparison groups associated with cap or mycelia has a significant number of DEGs (Figure 3D). The GO and KEGG enrichment analysis of DEGs between different tissue types showed the same functional DEG distribution trend (Figure 4; Supplementary Figure S1), confirming cap as the primary gene-expressing tissue during DI fruiting body formation. Earlier, Wang et al. (2020) identified 1954 unigenes across the developmental stages of DI *via* a *de novo* transcriptome study using peach-shaped and mature fruiting bodies. Meanwhile, we found far more DEGs in the cap of the DI fruiting body. Thus, the present study suggests that the cap is a tissue type that represents the expression pattern of DI fruiting bodies and plays a vital role in fruiting body differentiation.

### 3.3. Metabolome reveals novel resource value of DI tissues

The fruiting body of DI has a complex structure. Presently, the edible part of DI is the stipe, and the other four tissues are discarded. Therefore, we assessed the resource value of five DI tissues by analyzing the metabolite composition based on a widely-targeted metabolomic approach. We detected 729 metabolites from the five tissues, more than those (529) detected from the peach-shaped tissue of DI (Wang et al., 2021). In addition, we identified choline as the predominant metabolite in mycelia. Choline is essential for cell membranes’ structural integrity and signaling functions (Zeisel, 2006; Wiedeman et al., 2018). Studies have also detected choline in other medicinal fungi, such as Morchella (Yang et al., 2021), *Poria cocos* (Sun, 2014), and *Sanghuangporus baumii* (Zheng et al., 2021); however, it is usually extracted from the fungal fruiting bodies, unlike mycelium in the present study. Thus, the present study’s findings suggest that DI mycelium may be a potential raw material for extracting the medicinal ingredient after fermentation. We also found monosaccharides as the major metabolites in the primary edible tissue of DI, the stipe, suggesting presence of polysaccharides (Table 3). Polysaccharides are active components with antioxidant capacity and medicinal use (Liu et al., 2017). The present study also detected fungal polysaccharides as the main medicinal components of the edible fraction of DI, confirming the importance of DI stipe in exploiting fungal polysaccharides.

Additionally, we detected terpenoids in five tissue types of DI. Terpenoids are important medicinal components of higher fungi with promising economic value (Xiao and Zhong, 2016). Genome annotation based on the antiSMASH database identified 64 terpenoid synthesis related gene clusters (Supplementary Table S3), and metabolome analysis identified 12 kinds of terpenoids. Terpenoid biosynthetic genes and corresponding metabolites identified in the present study provide a reference for developing terpenoids from DI. Interestingly, we found that Dendronobilin I-iso1, the major terpenoid of DI, was mainly distributed in the volva but not in the stipe, the main edible part of DI. The sesquiterpenoid dendronobilin, a medicinal ingredient usually extracted from the Dendrobium genus and found in the traditional Chinese herbal medicine “Shi Hu,” has antitumor, antimutagenic, and immunomodulatory activities (Zhang et al., 2007; Meng et al., 2017). Therefore, our study suggests the development of terpenoid components from DI volva, previously considered agricultural waste. Taken together, the present study proposes a novel value of DI and promotes the development of mycelia and volva as resources for medicinal use.

### 3.4. Tryptophan metabolism in regulating DI fruiting body differentiation

Understanding the factors controlling fruiting body formation helps improve the quality and yield of DI through manual intervention. The transcriptome study revealed that mycelia and cap are the key parts affecting DI fruiting body differentiation (Figure 3). Further comparative metabolome analysis (mycelia vs. cap) revealed the importance of tryptophan metabolism in regulating DI fruiting body differentiation (Figure 7). Earlier, Yan et al. found that in *Lentinula edodes*, tryptophan metabolism affects fruiting body development (Yan et al., 2021), while Kück et al. reported that disrupting the signaling of amino acids, including tryptophan, delayed *Sordaria macrospora* fruiting body development (Kück, 2005). Our study also found that glycolytic metabolism occurs post tryptophan metabolism in DI (Figure 9). Thus, we infer that tryptophan metabolism may help extract energy from the vegetative mycelia during fruiting body formation, and this hypothesis is worth testing in the future. Moreover, we found specific enrichment of IAA in the cap during DI tryptophan metabolism. The plant growth hormone IAA is classified as an indole derivative of the auxin family (Nutaratat et al., 2016). It is one of the metabolites produced by rhizobia to promote plant growth (Sun et al., 2018). Therefore, our study suggests that the cap of DI, so far used as agricultural waste, has the potential to be developed into fertilizer to promote plant growth, and this requires detailed investigation.

### 3.5. Metabolite synthesis related genes affect DI fruiting body differentiation

Multi-omics study revealed that the distribution of metabolites in DI tissues was not consistent with the trend observed in gene expression (Figures 10A,B), probably because gene expression is a phased event, while metabolites have the property of accumulation. However, we identified through O2PLS analysis that the top 10 metabolites and DEGs with the most significant influence correlated with each other (Figures 10C,D; Supplementary Tables S6, S7). However, little is known about the function of these metabolites and genes; therefore, we hope to elucidate their relationship with DI fruiting body differentiation in future studies. In addition, we found that four metabolites of the tryptophan metabolic pathway, including IAA, were associated with DEGs identified by transcriptome sequencing, which indicates novel ways to regulate IAA synthesis. We identified three novel genes (Figure 10F; 5965_g, 5966_g, and 6182_g) related to IAA synthesis. The genes 5966_g and 6182_g were annotated as hypothetical proteins in the NR database, indicating a novel pathway to regulate endogenous IAA synthesis in plants; however, this needs to be investigated using model crops, such as Arabidopsis. Meanwhile, the gene 5966_g is associated with aldehyde dehydrogenase. A previous study reported that an aldehyde dehydrogenase from the bacterial plant pathogen Pseudomonas syringae produces IAA (Koren et al., 2017), which also suggests the significance of our finding. These genes may be valuable resources for improving plant growth.

## 4. Materials and methods

### 4.1. Materials

The DI variety Gutian-1 obtained from the Guangxi Academy of Agricultural Sciences, Nanning City (Guangxi, China), was used in this study. The material used for *de novo* genome sequencing was derived from a binucleated mycelial strain of fruiting body of Gutian-1, named ZS. Materials of five tissue types (Figure 1A) used for transcriptome and metabolome analyses (Figure 1B) were obtained from the production site of Guangxi Dan Gui Xian Agricultural Technology Co. Ltd., Guilin City (Guangxi, China). All materials were frozen in liquid nitrogen immediately after sampling and stored in a freezer at-80°C since Aug. 2021.

### 4.2. Methods

As shown in Figure 1C, we performed *de novo* sequencing of the DI genome. Then, we performed transcriptome and metabolome analyses using the five DI tissues. Finally, based on the multi-omics approach, we elucidated the molecular mechanism of DI fruiting body differentiation and the metabolites of importance in the different tissues.

#### 4.2.1. *De novo* genome sequencing

Genomic DNA was extracted from 0.1 g of ZS fresh mycelium using MabioFungal DNA Extraction Mini Kit B (Guangzhou, China), following the manufacturer’s instructions. The next-generation sequencing (NGS) libraries were generated from the extracted genomic DNA using NEB Next® Ultra™ DNA Library Prep Kit for Illumina® (NewEngland Biolabs, USA), following the manufacturer’s recommendations, and sequenced on a Novaseq6000 platform to obtain 150 bp paired-end reads. Meanwhile, genomic DNA was fragmented with g-TUBES (Covaris, USA) and end-repaired to prepare fragments with size >20 Kb. These DNA fragments were enriched by BluePippin size selection (Sage Science, USA), and the sequencing libraries were prepared using the SQK-LSK109 Sequencing Kit (Oxford Nanopore Technologies, Oxford Science Park, OX4 4DQ, UK), following the manufacturer’s protocol. Finally, the libraries were sequenced on a Nanopore MinION platform at the Guangdong Magigene Biotechnology Co., Ltd. (China).

The Illumina raw data and Nanopore raw data were filtered by fastp (v0.21) (Chen et al., 2018) and quast (Gurevich et al., 2013), respectively, using default parameters. The filtered reads were assembled to generate contigs without gaps using Canu (v1.8) (Koren et al., 2017). The hierarchical genome assembly process (HGAP) pipeline was used to correct for random errors in the long seed reads (seed length threshold 6 Kb) by aligning shorter reads from the same library against them with Pilon (v1.23) (Walker et al., 2014). Then, we used SSPACE (v1.1) (Boetzer et al., 2011) with preassembled reads for the final *de novo* assembly. GeneMark-ES (v4.69) (Lomsadze et al., 2014) and Augustus (v2.7) (Stanke et al., 2008) were used to retrieve the related coding genes in the DI genome. BUSCO (v5.4.3) analysis was used to assess the genome integrity (Manni et al., 2021).

We then used both software and merged result from GeneMark-ES and Augustus to obtain the final list of coding genes. Then, the gene functional annotation was carried out using GO (Gene Ontology; (Gene Ontology Consortium, 2021), KEGG (Kyoto Encyclopedia of Genes and Genomes) (Kanehisa and Goto, 2000), and KOG (Eukaryotic Orthologous Groups) (Tatusov et al., 2003). Meanwhile, the secondary metabolism-associated gene clusters were identified using antiSMASH^1^ (Blin et al., 2021) with the default parameters, and FCPD^2^ (Park et al., 2008) with the parameters of e-value <1e-10 and identity >40%. The Carbohydrate-Active enzymes (CAZymes) were predicted for the genome using dbCAN (Yin et al., 2012), with an e-value ≤1e-5.

#### 4.2.2. Transcriptome sequencing

Total RNA was extracted from 0.1 g of fresh DI tissues (stipe, cap, indusium, volva, and mycelium) using TRIzol reagent (Invitrogen, Carlsbad, CA, USA) according to the manufacturer’s protocol and sequenced on an Illumina Novaseq6000 system at the Gene Denovo Biotechnology Co. Ltd. (Guangzhou, China).

The raw reads were filtered to obtain high-quality reads using fastp (v0.18.0), which removed reads containing adapters, more than 10% unknown nucleotides (N), and more than 50% low-quality bases (*Q*-value ≤20) (Chen et al., 2018). These filtered reads were then aligned to the reference genome generated in this study. An index of the reference genome was built, and paired-end clean reads were mapped to the reference genome using HISAT (v2.2.4) (Kim et al., 2015) with “-rna-strandness RF” and other default parameters. The mapped reads of each sample were assembled using StringTie (v1.3.1) (Pertea et al., 2015, 2016). An FPKM (fragment per kilobase of transcript per million mapped reads) value was calculated for each transcription region to quantify the expression of genes and variations among different comparison groups using RSEM (v1.3.3) (Li and Dewey, 2011). Finally, DESeq2 (v1.36) (Love et al., 2014) was used to analyze the differential expression of genes between two groups and(Li et al., 2021) between two samples. The genes with a false discovery rate (FDR) <0.05 and an absolute fold change ≥2 were identified as the differentially expressed genes (DEGs). Venn analysis was performed to compare the DEGs between the different comparison groups using the VennDiagram package (v1.6.16) in R (Chen and Boutros, 2011).

Further, GO (Gene Ontology Consortium, 2021) and KEGG (Kanehisa and Goto, 2000) enrichment analyses were performed for the DEGs. The DEGs associated with specific GO terms and KEGG pathways compared to the genome background were filtered using the FDR ≤ 0.05 threshold. Finally, the Pearson’s correlation between DEGs of each tissue type was analyzed using the OmicShare tools^3^ to assess the reliability of the experimental results and the operational stability. A correlation coefficient closer to 1 indicates better repeatability between two experiments. Meanwhile, the correlation coefficient between two replicas was calculated to evaluate the repeatability between samples.

#### 4.2.3. Widely-targeted metabolome analysis

A widely-targeted metabolome analysis based on ultra-high-performance liquid chromatography-electrospray ionization-tandem mass spectrometry (UHPLC-ESI-MS/MS) was performed to identify the metabolites and their differences among the five tissues of DI at the Metware Biotechnology Co., Ltd. (Wuhan, China), as described earlier (Li et al., 2021). The DI different tissue samples were freeze-dried for 48 h and ground into powder. Approximately 100 mg of the powder was extracted with 70% aqueous methanol (0.6 ml), and the extract was analyzed on a UHPLC-ESI-MS/MS system (UHPLC, Shim-pack UFLC SHIMADZU CBM30A system, Kyoto, Japan; MS, Applied Biosystems 4,500 Q TRAP, Framingham, MA, USA). Three biological replicates were maintained for each tissue. Meanwhile, all the sample extracts were mixed to prepare the quality control (QC) sample used to test the measurement accuracy after every six samples.

The qualitative analysis of the primary and secondary mass spectrometry data was performed using a self-built database MWDB (v2.0; Metware Biotechnology Co., Ltd. Wuhan, China) and the publicly available databases, such as MassBank^4^, HMDB (Human Metabolome Database^5^), and METLIN^6^. Meanwhile, the quantitative analysis of the metabolites was performed using the multiple reaction monitoring mode (MRM) of triple quadrupole mass spectrometry. MultiQuant (v3.0.2) was used to access the mass spectrometry files and to integrate and correct the peaks. The area of each chromatographic peak represented the relative content of the metabolite; the mass spectra were integrated and corrected to determine the content of each metabolite in the different samples. Further, the levels of each metabolite in the various samples were compared based on the retention time and peak pattern.

The raw data were processed using the Analyst 1.6.3 software (AB Sciex, Framingham, MA, USA). The original abundance of the metabolites was log-transformed to normalize the data and decrease the variance. Principal component analysis (PCA), cluster analysis, and orthogonal projections to latent structures-discriminant analysis (OPLS-DA) were conducted for the metabolite data in R^7^, following the previously described methods (Li et al., 2021). Variable importance in projection (VIP) values of all metabolites from the OPLS-DA were extracted using the first component. Finally, the differential metabolites among the pairwise comparisons of different DI tissue types (such as mycelia vs. cap) were screened based on the following criteria: (i) VIP ≥ 1 (high confidence in pairwise comparisons); (ii) a fold change ≥2 and ≤ 0.5. Further, KEGG annotation and metabolic pathway analysis were performed for the differential metabolites. A hypergeometric test was used to identify the significantly enriched pathways (*p* < 0.05).

#### 4.2.4. Multi-omics analysis

Unsupervised principal component analysis (PCA) of transcriptome and metabolome data was performed using the statistics function prcomp within R^8^ after scaling the data. Two-way orthogonal partial least square (O2PLS) analysis was also performed to screen the genes and metabolites with a strong influence on DI tissue differentiation with R (OmicsPLS v1.2.0) (Bouhaddani et al., 2018). Further, the correlation between the transcriptome and metabolome data was analyzed by calculating the Pearson correlation coefficient in R (v4.7, base package), and the correlation network of genes and metabolites was built in R (igraph v1.3.4^9^).

#### 4.2.5. Quantitative polymerase chain reaction

Total RNA was extracted from five DI tissue types using TRIzol reagent (Section 2.2.2) and reverse transcribed (2 μg of RNA with an OD260/OD280 of 1.9–2.0) into cDNA using the iScript cDNA Synthesis Kit (Bio-Rad). Real-time quantitative PCR to validate the expression of five genes in the transcriptome data was performed with SYBR Green Master Mix (Thermo Fisher Scientific, MA, United States) on a QuantStudio™ Flex Real-Time PCR System, maintaining three technical repeats per sample. The housekeeping gene (reference genes) 5711_t was used to normalize mRNA expression levels, and the fold change in expression levels was defined using the 2^−ΔΔ*Ct*^ equation. The melting curve for each gene was generated to validate the specificity of the amplicon. The qPCR primers were designed using Primer Premier 5 and are listed in Supplementary Table S9.

## 5. Conclusion

The present study reveals the DI reference genome for further functional research. Transcriptome and metabolome analyses of the four tissues of the DI fruiting bodies and mycelium revealed the mechanism underlying DI fruiting body differentiation. However, future studies should aim to generate high-quality genomes by mononuclear sequencing. Our analysis also detected new metabolites from the mycelium (choline) and volva (dendronobilin) that expand the economic, medicinal, and agricultural values of DI. Additionally, unraveling the significance of tryptophan metabolism and novel genes related to IAA synthesis in regulating fruiting body formation proposes candidates for manipulating DI. The study thus raises new scientific questions on the developmental use of DI.

## Data availability statement

The datasets presented in this study can be found in online repositories. The names of the repository/repositories and accession number(s) can be found at: https://www.ncbi.nlm.nih.gov/, PRJNA877048 and JAQFWX000000000.1.

## Author contributions

MD, ZW, ZC, and LW contributed to conception and design of the study. MD, SL, XW, BF, SQ, YL, XL, CL, CZ, YY, JW, and FZ performed investigation. MD and SL performed the statistical analysis. MD wrote the first draft of the manuscript. All authors contributed to the article and approved the submitted version.

## Funding

This work was jointly funded by the National Natural Science Foundation of China (grant number NNSFC 32260715), Central Government Guides Local Funds for Science and Technology Development (grant number GuiKe ZY21195033), Guangxi Major Science and Technology Project (grant number GuiKe AA22117004), Guangxi Science and Technology Base and Special Talent (grant number GuiKe AD20297130), Science and Technology Pioneer Special of Guangxi Academy of Agricultural Sciences (grant number GuiNongKeMeng 202203-1-2), and Team Project for Guangx Academy of Agriculture Sciences (grant number Guinongke 2021YT004).

## Conflict of interest

The authors declare that the research was conducted in the absence of any commercial or financial relationships that could be construed as a potential conflict of interest.

## Publisher’s note

All claims expressed in this article are solely those of the authors and do not necessarily represent those of their affiliated organizations, or those of the publisher, the editors and the reviewers. Any product that may be evaluated in this article, or claim that may be made by its manufacturer, is not guaranteed or endorsed by the publisher.

## Footnotes

^1^ antismash.secondarymetabolites.org

^2^ http://p450.riceblast.snu.ac.kr/index.php?a=view

^3^ https://www.omicshare.com/tools

^4^ http://www.massbank.jp

^5^ http://www.hmdb.ca

^6^ http://metlin.scripps.edu/index.php

^7^ http://www.r-project.org/

^8^ www.r-project.org

^9^https://CRAN.R-project.org/package=igraph, access on 2022.08.18
