# Supplementary figures and images for: Genome, transcriptome, and metabolome analyses provide new insights into the resource development in an edible fungus Dictyophora indusiata
Source: Front Microbiol. 2023 Feb 9;14:1137159. doi: 10.3389/fmicb.2023.1137159 (PMC9948255; doi:10.3389/fmicb.2023.1137159)

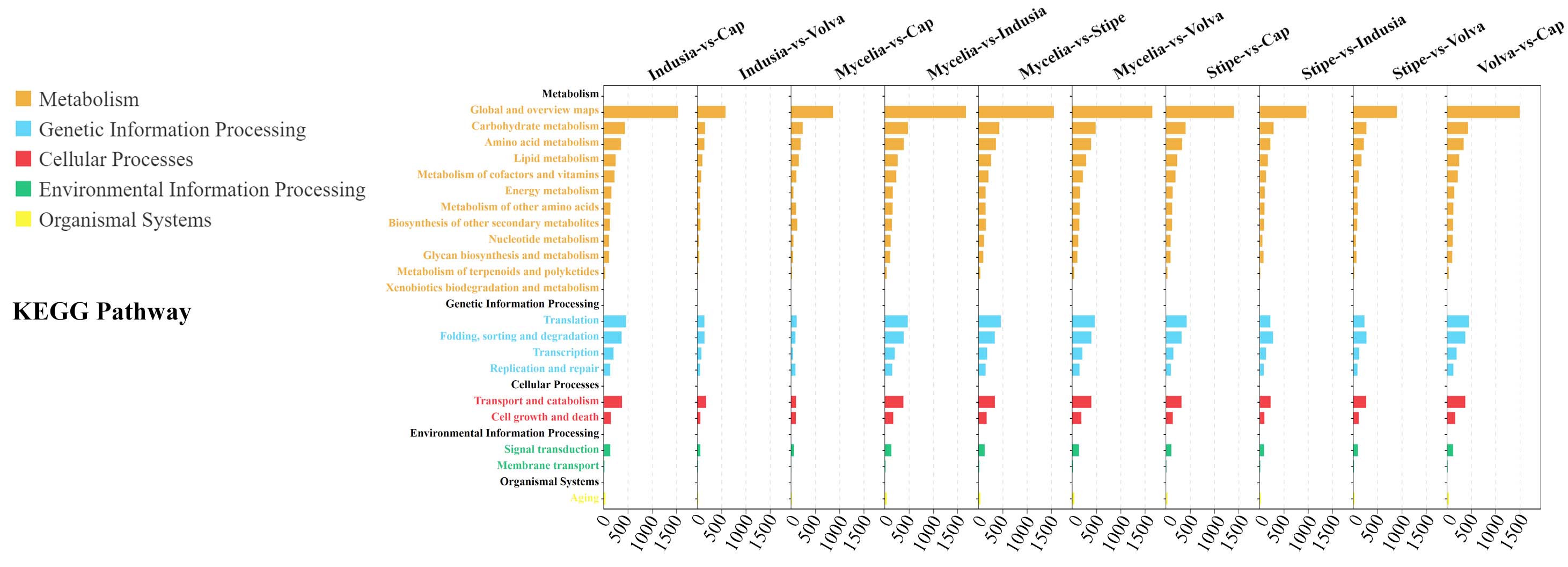

Supplement: Supplementary file 2 [file Image_1.JPEG]
